# Pregnancy induced Cushing’s syndrome and primary aldosteronism: a case report

**DOI:** 10.1186/s12884-020-03117-1

**Published:** 2020-07-25

**Authors:** Maria Kersten, Katharina Hancke, Wolfgang Janni, Katrina Kraft

**Affiliations:** 1 Department of Obstetrics and Gynecology, University of Ulm (Universitätsklinikum Ulm), Ulm, Germany; 2 Department of Obstetrics and Gynecology, Munich Klinik Harlaching (München Klinik Harlaching), Munich, Germany

**Keywords:** Cushing’s syndrome, Primary aldosteronism, Pregnancy, Metyrapone, Preeclampsia, Spironolactone

## Abstract

**Background:**

First manifestation of Cushing’s syndrome during pregnancy is rare. The diagnosis of both Cushing’s and primary aldosteronism within a pregnancy has not been previously documented. Diagnosis is especially challenging due to the normal physiological changes that occur during pregnancy. Consequently, many tests that are normally used for diagnosis are not reliable. Tumor based etiologies can be surgically removed. Etiologies that are not tumor based are challenging to treat during pregnancy.

**Case presentation:**

A 25 year old G1P0 was admitted in the 22 ^5/7^ week of pregnancy with elevated blood pressure (200/100 mm Hg), acne, moon facies, abdominal striae and hirsutism. With five antihypertensive medications her blood pressure remained 190/100 mm Hg. The patient was admitted to the ICU for intravenous medications and monitoring. She was diagnosed with Cushing’s syndrome and primary aldosteronism. In spite of therapy with spironolactone and metyrapone she developed preeclampsia and was delivered in the 26 ^0/7^ week of pregnancy. At her follow up visit eight weeks postpartum she had blood pressure within normal limits, no clinical signs or symptoms, and all medications had been discontinued.

**Conclusions:**

Early diagnosis of pregnancy induced Cushing’s syndrome and primary aldosteronism requires an interdisciplinary approach. Late detection has been associated with increased perinatal morbidity and mortality including but not limited to placental abruption and intrauterine demise. Collaboration is essential in the optimization of maternal and fetal outcomes.

## Background

First manifestation of Cushing’s syndrome (CS) and primary aldosteronism (PA) during pregnancy is rare. The most frequent etiologies have been attributed to adenomas, adrenal hyperplasia, or tumors. A small number of cases have been classified as pregnancy induced.

Due to the small number of documented cases of pregnancy induced CS there is not a lot known about the pathophysiology. Case reports of tumor free patients with adrenocorticotropic hormone (ACTH) -independent pregnancy induced CS have been published. It is hypothesized that some adrenocortical cells have estrogen receptors. These receptors are stimulated in pregnancy (as a result of elevated estrogen levels) resulting in an increase in cortisol production [1].

Diagnosis of CS and PA is challenging due to the normal physiological changes that occur in pregnancy. Many of the available diagnostic tests can be inconclusive. Brue et al. described the difficulty of diagnosing CS during pregnancy. The overlapping symptoms that occur as a result of hypercortisolism and the normal symptoms of pregnancy, including hirsutism, fatigue and weight gain can lead to a delay in diagnosis [2]. Signs and symptoms of CS such as hypokalemia may result from hyperemesis. Hypertension and hyperglycemia can be complications of pregnancy without underlying pathology.

Increase in plasma cortisol begins in the first trimester and continues to increase throughout the entire pregnancy. There are elevations in corticosteroid-binding globulin (CBG) and increases in CRH and ACTH produced by the placenta (beginning at week 7 and increasing until the end of pregnancy). The increased total levels of cortisol can make the interpretation of tests, such as the dexamethasone suppression test, extremely difficult. Urinary free cortisol (UFC) increases during the second trimester. Diagnostic tests with UFC are only reliable in the first trimester of pregnancy. CS that is diagnosed during pregnancy has been associated with more complications than CS diagnosed outside of pregnancy. Additional complications include growth retardation, preeclampsia, premature delivery, and intrauterine fetal demise [2]. PA symptoms include hypertension and hypokalemia. The incidence of preeclampsia in patients with PA is not higher than that of the general population [3].

We present a case of a patient with pregnancy induced CS and PA.

## Case presentation

**Fig. 1:**
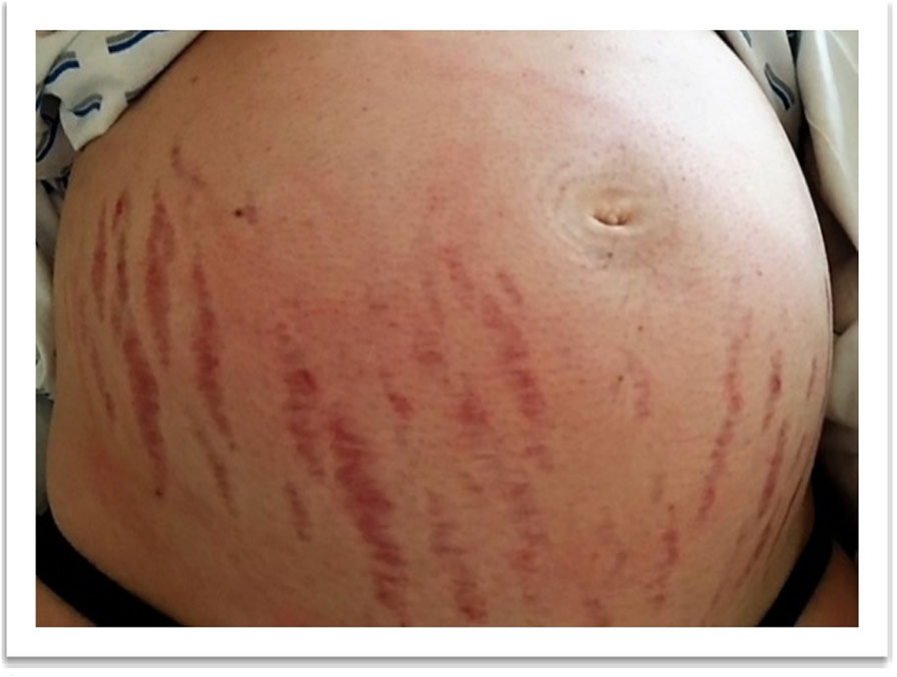
Typical signs of Cushing's syndrome including facial acne, moon facies, and abdominal striae

**Fig. 2:**
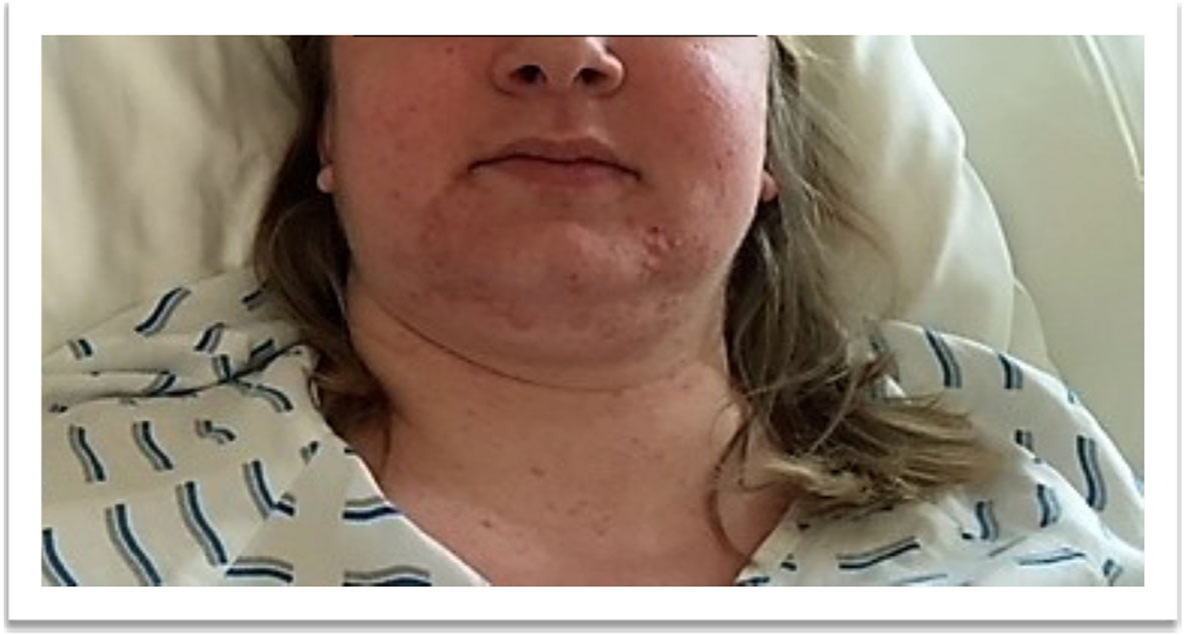
Typical signs of Cushing's syndrome including facial acne, moon facies, and abdominal striae

A 25 year old G1P0 was transferred to our center in the 22^5/7^ week of pregnancy. The patient was admitted with blood pressure 200/100 mm Hg and presented with acne, moon facies, purple abdominal striae and hirsutism that she first noticed five weeks before admission (Figs. 1 and 2). She denied headache, abdominal pain, edema, visual disturbances, or rapid weight gain. Reflexes were normal. Sonography on admission showed singleton pregnancy with growth at the 68th percentile, fetal and maternal Doppler within normal limits. The patient denied family history of pregnancy-induced illnesses including CS, hypertension in pregnancy, or a history of preeclampsia.

We initiated therapy with methyldopa and began additional medications including hydralazine 25 milligrams (mg) three times daily (TID), metoprolol 95 mg two times daily (BID), nifedipine (retard) 20 mg BID, hydrochlorothiazide (HCTZ) 12.5 mg BID. The methyldopa was maximized at 2000 mg daily. In spite of the five antihypertensive medications her blood pressure remained elevated at190/100 mm Hg. The patient was transferred to our intensive care unit where the oral medications were supplemented with intravenous urapidil (alpha 1 adrenoreceptor antagonist and 5-HT_1A_ receptor agonist). Hydralazine was given intravenously.

Pheochromocytoma was ruled out (free metanephrine 18.5 nanogram/Liter (ng/L (normal 7.9–88.7)), free normetanephrine < 2.8 ng/l (normal 20.1–135.4)). A negative renal artery Doppler allowed us to rule out stenosis. Magnetic resonance imaging (MRI) to rule out adrenal adenoma was inconclusive. Micronodules in the left adrenal gland (the largest 4 millimeter) were found so that unilateral adrenal hyperplasia could not be completely ruled out. After consultation with our urology department, surgical intervention was not warranted.

MRI of the neck and thorax were negative for tumors. In 24^1/7^ weeks we saw an increase in aspartate aminotransferase (AST) to 74 U/l and in alanine aminotransferase (ALT) to 102 U/l (normal < 50 U/l and < 45 U/l respectively) with a decrease in thrombocytes to 89 Giga/l (normal 150–450 Giga/l) and a decreasing haptoglobin to 0.41 g/l (normal 0.3–2 g/l).

The patient began rapidly gaining weight (13 kg within 13 days).

Due to the complex clinical presentation we consulted with our endocrinology department and performed additional diagnostic tests. Plasma cortisol levels were found to be elevated at 50.3 mcg/dl, (normal 2.47–19.5 mcg/dl), urinary free cortisol (UFC) was elevated at 974 µg/day (mcg/d, normal 50–190 mcg/d), ACTH was low at 3.6 pg/ml (normal 7.2–63.3), and the aldosterone-to-renin ratio (ARR) was elevated at 100.1 (normal 0.5–37.8). A summary of the hormonal results can be found in Table 1.

**Table 1:** Hormonal findings in chronological order

	23 ^0/7^ weeks	24 ^4/7^ weeks	25 ^3/7^ weeks	25 ^5/7^ weeks	1 month after delivery	2 months after delivery	4 months after delivery
Plasma cortisol (normal 2.47–19.5 mcg/dl)	50.3		36.4	25.5	15.4	4.65	
Urinary free cortisol (normal 50–190 mcg/d)		974					88.5
ACTH (normal 7.2–63.3 pg/ml)	3.6				14.4	23.7	
Aldosterone-to-renin ratio (normal 0.5–37.8)	100.1					65.7	23.6

A dexamethasone suppression test was performed and resulted in a cortisol of 39 mcg/dl (no suppression) and ACTH < 1.5 pg/ml. The patient was diagnosed with CS and PA and started on spironolactone 100 mg BID and metyrapone 2.5 g daily at 25^0/7^ weeks.

A risk of undervirilization has been described in male rat infants exposed to high doses of sprinolactone. To date undervirilization not been observed in humans [3]. The sex of the fetus (female) was determined and confirmed on multiple occasions prior to beginning the spironolactone therapy. Following consultation with our endocrinology and embryotoxicology departments and after reviewing the literature, we began therapy with spironolactone 100 mg twice daily.

Hypomagnesemia was treated intravenously after oral substitution attempts failed. After three days of therapy the intravenous urapidil and hydralazine could be discontinued.

The fetus was monitored for growth and fetal Doppler and cardiotocography were regularly performed.

In 26^0/7^ weeks an abnormal fetal Doppler with near zero flow in the ductus venosus, zero flow in the umbilical artery and low flow in the middle cerebral artery (MCA) was diagnosed. The patient was delivered by cesarean section on the same day. Antenatal corticosteroids were not given due to the elevated maternal cortisol blood levels.

We delivered a female newborn with birthweight 595 g (13th percentile), Apgar 4/7/10, umbilical artery pH 7.20, Base Excess − 4.7. The newborn was admitted to the neonatal intensive care ward and received surfactant postnatally. Hypoglycemia and hyponatremia were successfully treated. Our neonatologists were concerned about the high levels of maternal cortisol that were potentially transferred to the fetus in utero. The newborn had a significant risk for postnatal adrenal cortex insufficiency (due to potential in utero suppression) with subsequent hypocortisolism. A prophylactic hydrocortisone therapy was initiated to decrease the risk of a subsequent life threatening Addisonian crisis. Stress of delivery alone can lead to elevated cortisol levels in newborns after delivery. Neonatal cortisol levels remained elevated on the second day of life. The hydrocortisone therapy could be reduced and eventually discontinued on day 8 of life.

There were no further complications, no evidence of bronchopulmonary dysplasia, intraventricular hemorrhage or necrotizing enterocolitis. The newborn was discharged without long term medications or complications.

The patient was discharged with metyrapone 2.5 g daily, spironolactone 100 mg BID, methyldopa 2000 mg daily, hydralazine 25 mg TID, metoprolol 95 mg BID, HCTZ 12.5 mg BID and nifedipine 5 mg BID. Within 2 months all medications could be discontinued, the blood pressure was within normal limits (110/70 mm Hg) and within 4 months all laboratory values were within normal limits (Table 1).

## Discussion and conclusions

CS and PA are rare diseases in pregnancy. Roughly 200 cases of CS and less than 50 cases of PA in pregnancy have been published [3, 4]. Published cases of simultaneous CS and PA in pregnancy could not be found.

Pregnancy induced CS is very rare. Kaperlik-Zaluska et al. described a patient who had signs and symptoms of CS in three pregnancies. The patient was reported to have hirsutism, acne, striae, and lab abnormalities consistent with CS. The first two pregnancies ended prematurely after placental abruption in the 24th and 25th weeks of pregnancy. The third pregnancy was monitored very closely and a therapy with metyrapone was started in the 6th week of pregnancy. At 28 weeks she developed hypertension, at 32 weeks she was delivered per emergency cesarean due to placental abruption [1]. Examinations performed after delivery and during follow up visits showed no evidence of CS, lab values were within normal limits, the clinical signs and symptoms diminished.

Close et al. described a case of CS due to bilateral adrenal hyperplasia found at 23 weeks of pregnancy. The patient was treated with metyrapone until 34 weeks and was delivered via cesarean section due to decline in fetal growth. Four weeks after delivery adrenal function was within normal limits [5].

Andreescu et al. reported cases of CS within pregnancy in which the increase in human chorionic gonadotropin hormone (hCG) was associated with hypercortisolism. Aberrant luteinizing hormone (LH) receptors were found within adrenal adenomas after adrenalectomy. These adenomas were responsive to luteinizing hormone releasing hormone (LHRH) and hCG, leading the authors to conclude that high levels of hCG resulted in high levels of cortisol with the associated signs and symptoms of CS [6]. The patients with aberrant receptors continued to have symptoms and abnormal lab values after delivery. After adrenalectomy the symptoms decreased and the lab values normalized. Pregnancy induced cases of CS are summarized in Table 2.

**Table 2:** Summary of pregnancy induced CS cases

Case	Presentation	Therapy and Imaging	Outcome
Kasperlik-Zaluska 2000 [1]	Hypertension (190/110 mmHg), muscular atrophy, purple striae, hirsutism, hypokalemia at 16 weeks	None described	Cesarean section 24th week due placental abruption, postpartum lab values normal after 4 weeks
Kasperlik-Zaluska 2000 [1]	Similar features with elevated UFC at 16 weeks	None described	Cesarean section 25th week due to placental abruption, postpartum lab values normal after 3 months
Kasperlik-Zaluska 2000 [1]	Elevated UFC at 5 weeks, no symptoms	Metyrapone 0.75 g daily until 17 weeks, then increased to 1 gram due to increasing cortisol	Cesarean section at 32nd week due to placental abruption, normalization of lab values beginning at 2 weeks postpartum
Close 1993 [5]	Elevated cortisol, UFC and CS at 23 weeks	Bilateral adrenal hyperplasia in CT, Metyrapone	Cesarean section at 34 weeks by growth retardation, normal lab values at 4 weeks postpartum
Andreescu 2017 [6]	Purple striae, bruising, hypertension (165/90 mmHg), mild hypokalemia, proteinuria at 32 weeks, elevated UFC	Enalapril 40 mg daily Labetalol 600 mg daily CT: 3.8 cm Adenoma, LH receptor positive (immunohistochemical examination)	Induction of Labor at 35 weeks, Adrenalectomy and hydrocortisone replacement 4 months postpartum
Andreescu 2017 [6]	Hypertension (140/86 mmHg), purple striae, hirsutism, diabetes mellitus	Labetalol 400 mg daily MRI: 3.3 cm Adenoma, LH receptor positive (immunohistochemical examination)	Spontaneous labor and vaginal delivery 38 weeks, Adrenalectomy and hydrocortisone around 4 months postpartum
Andreescu 2017 [6]	Gestational diabetes, depression, hirsutism, bruising	Paroxetine 20 mg daily CT: 3.4 cm Adenoma (no immunohistochemical examination performed)	Spontaneous labor and vaginal delivery at 38 weeks, Adrenalectomy and hydrocortisone 6 months postpartum

Micronodules were detected in our MRI but were not interpreted by our radiology department as adenomas. Unilateral adrenal hyperplasia could not be completely ruled out. It is possible that our patient had aberrant receptors without the presence of adenomas. In order to completely rule out the presence of aberrant receptors additional diagnostic tests would have to be performed postpartum including an in vivo acute hormone stimulation test [6]. Gene expression tests are definitive for the diagnosis, but require sampling of adrenal tissue.

The commonest causes of PA are aldosterone-producing adenoma and idiopathic adrenal bilateral hyperplasia. Symptoms are less severe than in CS and are often limited to hypertension. A retrospective study found no increased incidence of preeclampsia in patients with PA than that of the general population [3].

The diagnosis of CS in pregnancy is especially challenging due to the normal hormonal changes that occur. There is an increase in plasma cortisol, salivary cortisol and UFC in the first trimester. The levels increase continually throughout the pregnancy, resulting in plasma cortisol levels that are two to three times higher by the third trimester. These high levels make the dexamethasone suppression test difficult to interpret [2]. Brue et al. described the difficulty of diagnosing CS during pregnancy due to the overlapping of symptoms that occur as a result of hypercortisolism and the normal symptoms of pregnancy. The symptoms include hirsutism, fatigue and weight gain [2]. Hypokalemia may result from hyperemesis, and hypertension and hyperglycemia can be complications of pregnancy without underlying pathology. There are elevations in corticosteroid-binding globulin (CBG) and increases in CRH and ACTH produced by the placenta (beginning at week 7 and increasing until the end of pregnancy). The increased levels of cortisol make interpretation of the dexamethasone suppression test difficult. Urinary free cortisol (UFC) increases during the second trimester. Tests utilizing UFC are therefore considered to reliable only in the first trimester of pregnancy. With regard to salivary cortisol levels, Ambroziak et al. could not detect differences in the salivary cortisol levels during pregnancy. They recommended the utilization of normal adult levels for pregnant women despite reporting higher salivary levels in some pregnant patients with hypercortisolism [7]. In spite of these confounding results, salivary cortisol levels may still be helpful for diagnosis of hypercortisolism in the first two trimesters of pregnancy [2].

The renin-aldosterone axis changes during pregnancy. The increase in estrogen and placental renin combined with increased progesterone results in increased aldosterone and deoxycorticosterone. Despite these changes there are few signs and symptoms that are considered as “classic” indicators of hypercortisolism and include muscle weakness, weight gain, purple striae, and bruising [2]. Pregnant patients presenting with both hypokalemia and hypertension should be screened for PA. Diagnosis of PA in pregnancy is made in patients presenting with suppressed renin (normally elevated in pregnancy) and an elevated aldosterone-to-renin ratio (ARR). False negative results are possible. MRI and ultrasound are useful imaging modalities. Confirmatory tests such as saline infusion tests and captopril tests are contraindicated in pregnancy [3].

Treatment of CS and PA is challenging. While surgical management has often been described as superior to medical therapy, the evidence is limited to case reports. If surgery must be performed, the recommended time frame is during the second trimester [2].

Ketoconazole is widely used for treatment of CS in non-pregnant women but is contraindicated in pregnancy. There are isolated case reports of women who became pregnant while being treated with ketoconazole. In all cases the therapy was discontinued as soon as the pregnancy was confirmed.

There are reports of patients undergoing surgery in the second trimester and being treated postoperatively with metyrapone or hydrocortisone [8]. Metyrapone is a medication that has been used both successfully and unsuccessfully in the treatment of CS in pregnant patients. Classified as an inhibitor of glucocorticoid synthesis, the medication inhibits 11-beta-hydroxylase, thereby blocking the production of cortisol. This blockade results in increased ACTH, 11-deoxycortisol, and deoxycorticosterone. The increase in deoxycorticosterone has been known to worsen hypertension and has been therefore associated with an increased risk of preeclampsia [2]. Lim et al. performed a literature search in Pubmed and Medline and found a total of 12 patients with CS in pregnancies that were subsequently treated with metyrapone. An estimated 20% of these women developed pre-eclampsia. Two neonatal deaths and one stillborn delivery were reported [9]. Metyrapone passes through the placenta. Its effects on fetal adrenal steroid synthesis are not well documented.

In PA mineralcorticoid receptor antagonists are the treatment of choice. Some animal studies have reported an association between spironolactone and genital anomalies in males. These results could not be reproduced in human studies. Rose et al. published a study in which rats were given 400 milligrams daily. The undervirilization could not be confirmed [10]. An extensive analysis of the data was performed by Liszewski and Boull in April 2019. The authors examined the literature and found that feminization was reported in 6 of 9 animal studies. Five of the studies exposed the animals to doses of more than 200 mg daily. Five human cases where spironolactone was given during pregnancy were summarized; normal genitals at birth were reported in all cases [11].

A therapy with eplerenone would have been an alternative to spironolactone. Eplerenone is a medication that has been on the market for less than 20 years and carries an FDA Pregnancy Category B classification, making it preferential to medications with category C. There are not enough studies in pregnant women to make conclusions about its efficacy in comparison to spironolactone in the treatment of primary aldosteronism.

Brue described a fetal upregulation of 11-beta-hydroxysteroid dehydrogenase-type 2 in fetuses exposed to high maternal cortisol levels. In spite of this protective measure, pregnancies with CS have been documented to have increased incidences of prematurity (43%), growth retardation (21%), and intrauterine fetal demise (5%). Outcomes were significantly worse for mothers who were diagnosed during pregnancy [2].

The diagnosis of CS and PA in pregnancy is challenging. For patients without obvious tumor etiologies the treatment is limited to medications and can be difficult. The use of metyrapone has been widely documented. Its use is associated with the development of preeclampsia and its effects on the fetus and fetal steroid synthesis remain unclear. In PA mineralcorticoid receptor antagonists are the treatment of choice.

Combined CS and PA in a pregnancy are rare and extremely difficult to treat. Due to the substantial risk of delivery complications including premature delivery, placental abruption, and preeclampsia, patients should be referred to a perinatal center. Early diagnosis combined with an interdisciplinary approach is essential in the optimization of maternal and fetal outcomes.

## Data Availability

Not applicable.

## Abbreviations

CS: Cushing's syndrome
PA: Primary aldosteronism
ACTH: Adrenocorticotropic hormone
CBG: Corticosteroid-binding globulin
CRH: Corticotropin-releasing hormone
TID: Three times daily
BID: Two times daily
MRI: Magnetic resonance imaging
AST: Aspartate Aminotransferase
ALT: Alanine Aminotransferase
U/l: Units per liter
g/l: Gram per Liter
mcg/dl: Microgram per deciliter
mcg/d: Microgram per day
pg/ml: Picogram per milliliter
ARR: Aldosterone-to-renin ratio
MCA: Middle cerebral artery
HCTZ: Hydrochlorothiazide
hCG: Human chorionic gonadotropin hormone
LH: Luteinizing hormone
LHRH: Luteinizing hormone-releasing hormone
UFC: Urinary free cortisol
